# Herbal medicines for SOD1^G93A^ mice of amyotrophic lateral sclerosis: preclinical evidence and possible immunologic mechanism

**DOI:** 10.3389/fimmu.2024.1433929

**Published:** 2024-09-17

**Authors:** Jiang-Li Yang, Jing-Ying Wu, Jing-Jing Liu, Guo-Qing Zheng

**Affiliations:** Department of Neurology, The First Affiliated Hospital of Zhejiang Chinese Medical University (Zhejiang Provincial Hospital of Chinese Medicine), Hangzhou, China

**Keywords:** amyotrophic lateral sclerosis, herbal medicine, meta-analysis, scoping review, mechanism of immunology

## Abstract

Currently, there is no cure or effective treatment for Amyotrophic Lateral Sclerosis (ALS). The mechanisms underlying ALS remain unclear, with immunological factors potentially playing a significant role. Adhering to the Preferred Reporting Items for Systematic Reviews and Meta-Analysis (PRISMA), a systematic review of preclinical studies was conducted, searching seven databases including PubMed, covering literature from the inception of the databases to April 10, 2024. Methodological quality of the included literature was assessed using CAMARADES, while the risk of bias in the included studies was evaluated using SYRCLE’s ROB tool. Review Manager 5.4.1 statistical software was used for meta-analysis of the outcomes. The scoping review followed the Joanna Briggs Institute Methodological Guidelines and reporting of this review followed the PRISMA-extension for Scoping Reviews (PRISMA -ScR) checklist to explore the immunological mechanisms of Herbal Medicine (HM) in treating ALS. This systematic review and meta-analysis involved 18 studies with a total of 443 animals. The studies scored between 4 to 8 for methodological quality and 3 to 7 for risk of bias, both summing up to 10.A remarkable effects of HM in ALS mice, including onset time(Standardized Mean Difference(SMD): 1.75, 95% Confidence Interval(CI) (1.14 ~ 2.36), Z = 5.60, *P* < 0.01), survival time(SMD = 1.42, 95% CI (0.79 ~ 2.04), Z = 4.44, P < 0.01), stride length(SMD=1.90, 95% CI (1.21 to 2.59), Z = 5.39, P < 0.01) and duration time (Mean Difference(MD)=6.79, 95% CI [-0.28, 13.87], Z=1.88, P =0.06), showing HM’s certain efficiency in treating ALS mice. The scoping review ultimately included 35 articles for review. HMs may treat ALS through mechanisms such as combating oxidative stress, excitatory amino acid toxicity, and calcium cytotoxicity, understanding and exploring the mechanisms will bring hope to patients. Individual herbs and their formulations within HM address ALS through a variety of immune pathways, including safeguarding the blood-brain barrier, countering neuroinflammation, impeding complement system activation, mitigating natural killer cell toxicity, and regulating T cell-mediated immune pathways. The preclinical evidence supports the utilization of HM as a conventional treatment for ALS mice. Growing evidence indicates that HM may potentially delay neurological degeneration in ALS by activating diverse signaling pathways, especially immune pathways.

## 1 Introduction

Amyotrophic Lateral Sclerosis(ALS)is characterized by dysfunction of upper motor neurons and lower motor neurons, affecting the medulla, cervical, thoracic, and/or lumbar segments. This results in progressive weakening of voluntary skeletal muscles, leading to symptoms such as limb movement impairment, swallowing difficulty, speech problems (dysarthria), and respiratory dysfunction (1). The median survival time of ALS is reported as 20 to 48 months after the onset of symptoms, among which 90% to 95% are sporadic ALS, and 5% to 10% of patients are familial ALS (2). As ALS remains incurable, treatment is focused on using disease modifying therapies and maximizing quality of life (1). Some countries have approved riluzole and edaravone as medications for slowing the progression of ALS. Riluzole, an anti-glutamate agent, prolongs survival in ALS patients in clinical trials and post-marketing analyses, but whether this occurs in all stages of ALS or only in advanced disease remains controversial (3). Some studies have reported that people with ALS who meet certain criteria may benefit from the use of edaravone, which has antioxidant properties (4–6). However, possibly because the study design lacked general applicability to the wider population of ALS patients, post-marketing analyses have raised questions about the safety and benefits of edaravone. As a result, the use of edaravone is still controversial and does not yet have regulatory approval around the world (1).

An increasing number of people with ALS resort to HMs because of the modest benefits of current therapies. In China and several other Asian countries, HM is widely used alongside Western Medicine (WM), with both systems cooperating to provide healthcare services for the population. However, the diverse responses to HM continue to be a subject of ongoing debate and challenge (7). In recent decades, numerous studies have investigated the effectiveness of HMs in treating ALS. Previous systematic reviews have indicated that short-term adjunctive use of HM may improve ALS Functional Rating Scale (ALSFRS) scores and clinical outcomes, with a favorable safety profile compared to placebo or riluzole alone. However, further research is required to evaluate the long-term efficacy of patient-oriented outcomes (8). Additionally, there is very low to low-quality evidence indicating that HMs may produce superior treatment responses for ALS without an increased risk of adverse events (9). Nevertheless, with their widespread use, HMs have attracted both praise and criticism. A single-center cohort study found that certain HMs were associated with a poorer prognosis in ALS patients (10).

ALS is a fatal central nervous system neurodegenerative disease. At present, the etiology and pathogenesis of ALS are still unclear. Evidence from clinical studies suggests that dysregulated immune responses contribute to heterogeneity in the clinical presentation of ALS (11). Immune inflammation caused by abnormal immune disorders, such as microglial activation, astrocyte proliferation and T cell infiltration, can be observed at the site of motor neuron degeneration (12), and immune cell infiltration can accelerate disease progression (13), All these suggest that abnormal immune disorders play an important role in the occurrence and development of ALS, and this treatment direction will be an effective treatment strategy. Exactly, in animal models of ALS and *in vitro*, the effects of HMs have been consistently praised. This phenomenon creates confusion among researchers and patients regarding the efficacy of HM in ALS studies, leading to questions about its utility and study design implications (10). The efficacy and mechanisms of HMs for experimental ALS have not been systematically evaluated yet. In addition, preclinical systematic review of animal data can provide preclinical evidence for the potential translational value from animal models to human disease. Thus, the present study aims to evaluate the efficacy and immunologic mechanisms of HMs through experimental ALS animal models.

## 2 Methods

### 2.1 Systematic analysis

#### 2.1.1 Approach

We followed the guidelines outlined in the Preferred Reporting Items for Systematic Reviews and Meta-Analysis (PRISMA) (14). There was no need for ethical approval because this was a literature research.

#### 2.1.2 Data sources and search strategy

Two experienced researchers (YJL and WJY) independently carried out extensive searches for studies on HM for ALS. We searched the following electronic databases from their inception until April 10, 2024: PubMed, EMBASE, Web of Science, Cochrane Library, Wan fang database, Vasoactive Intestinal Polypeptide (VIP), China National Knowledge Infrastructure (CNKI), and Sinomed. The following keywords were used for the preclinical evidence: (“Chinese herbal medicine” OR “herbal medicine” OR “Traditional Chinese medicine” OR “Chinese Drug” OR “Korean Medicine” OR “East Asia Medicine”) AND (“Amyotrophic Lateral Sclerosis” OR “motor neuron disease” OR “Gehrig’s Disease” OR “Motor System Diseases”). Moreover, reference lists of potential articles were searched for relevant studies. All the studies included were limited on animals (**Supplementary Data Sheet 1**).

#### 2.1.3 Inclusion and exclusion criteria

Literature screening was conducted collaboratively by a minimum of two members of our research team. In the initial screening phase, a comprehensive plan for full-text screening was meticulously devised. Each researcher independently assessed the abstracts and methodologies of the literature, initially selecting relevant articles. Subsequently, selected literature underwent thorough full-text review, with articles meeting the criteria being ultimately chosen. The screening results were then integrated and consolidated by the research team members, leading to the creation of corresponding flowcharts. In cases of differing opinions among team members during the screening process, careful negotiation and discussion were undertaken to reach a unanimous final decision.

The studies meeting the inclusion criteria were included in the meta-analysis: (1) Studies of HMs for ALS; (2) Inclusion of studies with Riluzole, Edaravone, normal saline and distilled water as control groups; (3) HM as monotherapy was used in the intervention group; (4) Identified SOD1^G93A^ transgene mouse; (5) The primary outcome measures were onset time or survival time, while the secondary outcome measure was stride length and duration time. When the mice on the transfer bar movement for the longest time less than 5 min record the day as the onset time (15). After mice will lie down if it cannot turn to normal within 30 seconds gesture, determine its death, the death date of record this day for mice (16).

Exclusion criteria for the studies were as follows: (1) Lack of a control group; (2) Case reports, clinical experiments, reviews; (3) Cell experiments; (4) Repeated publications or studies with missing data.

#### 2.1.4 Data extraction

Two separate authors independently extracted the following details from the included studies: (1) The primary author’s name and the year of publication; (2) The specific details of animals for each study, including species, quantity, gender; (3) Accepted ALS mouse model; (4) The specifics of the treatment group, including the dosage, administration method of the therapeutic drug, treatment duration, and corresponding details for the control group; and (5) Efforts were made to contact authors for supplementary information when certain publications contained only graphical data. In instances of no response, numerical data were extracted from graphs using digital ruler software.

#### 2.1.5 Study quality and risk of bias

In the methodology section of this study, we will employ two evaluation tools, CAMARADES (Collaborative Approach to Meta-Analysis and Review of Animal Data from Experimental Studies) (17) and SYRCLE’s ROB tool (Systematic Review Centre for Laboratory animal Experimentation’s Risk of Bias) (18), to assess the quality and risk of bias in the included studies. CAMARADES will be used to evaluate the experimental design and methodological quality of the studies, such as sample size, randomization, and blinding. SYRCLE’s ROB will be used to assess the risk of bias in each study during the experimental process, including selective reporting and sample size calculation. By utilizing these two evaluation tools in conjunction, we aim to comprehensively assess the quality and risk of bias in animal experimental studies, providing stronger support for the interpretation and generalization of research results.

CAMARADES primarily focused on ten aspects of the literature: (1) Publication in a peer-reviewed journal; (2) statement of temperature control; (3) randomization to treatment group; (4) allocation concealment; (5) blinded assessment of outcome; (6) avoidance of anesthetics with known notable intrinsic neuroprotective properties; (7) Use appropriate ALS animal models; (8) sample size calculation; (9) compliance with animal welfare regulations; (10) declared any potential conflict of interest. The scoring method of this scale involves assigning scores on a scale ranging from 0 to 10.

In contrast, SYRCLE’s ROB primarily focused on ten aspects of the literature: (1) Randomization (selection bias); (2) Random sequence generation (selection bias); (3) Baseline characteristics (selection bias); (4) Allocation concealment; (5) Random housing (performance bias); (6) Blinding of personnel (performance bias); (7) Random outcome assessment (detection bias); (8) Blinding of outcome assessment (detection bias); (9) Incomplete outcome data (attrition bias); (10) Selective reporting (reporting bias). According to the scoring method of this scale, scores are assigned on a scale from 0 to 10.

#### 2.1.6 Statistical analysis

Review Manager 5.4.1 statistical software was used for meta-analysis of the included literature. Stata 15 software is used to conduct sensitivity analysis to assess the robustness of data analysis. The outcome measures selected in our study include: onset time, survival time, stride length, and disease duration. All outcome measures are continuous. For these variables, we applied Weighted Mean Difference (WMD) or Standardized Mean Difference (SMD) as aggregate statistics. Each effect size included a 95% confidence interval (95% CI), and a combined p-value ≤ 0.05 was deemed statistically significant.

### 2.2 Scoping review

#### 2.2.1 Approach

The scoping review adhered to the Joanna Briggs Institute Methodological Guidelines (19) and reporting of this review followed the Preferred Reporting Items for Systematic Reviews and Meta-Analysis extension for Scoping Reviews (PRISMA-ScR) checklist (20).There was no need for ethical approval because this was a literature research.

#### 2.2.2 Data sources and search strategy

Two experienced researchers (YJL and LJJ) independently conducted comprehensive searches for studies on the immune mechanism effects of HM, using the PubMed and CNKI databases from their inception until April 10, 2024. The following keywords were used for the possible mechanisms of immunology: (“Chinese herbal medicine” OR “herbal medicine” OR “Traditional Chinese medicine” OR “Chinese Drug” OR “Korean Medicine” OR “East Asia Medicine”) AND (“immune response” OR “immune dysregulation” OR “immunologic mechanism” OR “Immunological reaction” OR “Immune Processes”). both in English and Chinese. There is no limitation on language or publication type. We also screened the references of included studies to ensure that no eligible studies were missed by the search strategy (**Supplementary Data Sheet 2**).

#### 2.2.3 Inclusion and exclusion criteria

In our study, two team members (YJL and WJY) collaborated on literature screening. We meticulously planned the full-text screening process. Each researcher independently evaluated abstracts and methodologies to find relevant articles. Selected literature underwent a rigorous full-text review, advancing only if meeting our criteria. We synthesized the screening results and illustrated them with concise flowcharts. In case of disagreements, we discussed until reaching a consensus to ensure the integrity of our decisions.

For inclusion in the scoping review, articles must focus on the mechanisms of HM in treating ALS, with a particular emphasis on immune mechanisms. The search will encompass all types of qualitative, quantitative, and mixed-method studies, irrespective of their design, and will not be restricted by language. Publications were excluded if they did not discuss the results of ALS or HM. Additionally, publications focusing solely on WM treatment and not on the mechanism of herbal treatment for ALS were excluded. Finally, publications meeting the aforementioned criteria but reporting non-primary research, such as editorials, letters, concept papers, review articles, unpublished literature, dissertations, books, and book studies, were also excluded.

#### 2.2.4 Data extraction

We collected data using specially designed extraction forms. The following information was recorded for each study: (a) author, (b) year of publication,(c) journal, (d) country, (e) type of studies, (f) Involved mechanisms (g)Involved HMs Two researchers performed the data extraction and synthesis processes independently (YJL and LJJ). A third researcher resolved any disagreement.

## 3 Results

### 3.1 Systematic analysis

#### 3.1.1 Study inclusion

**Figure 1:**
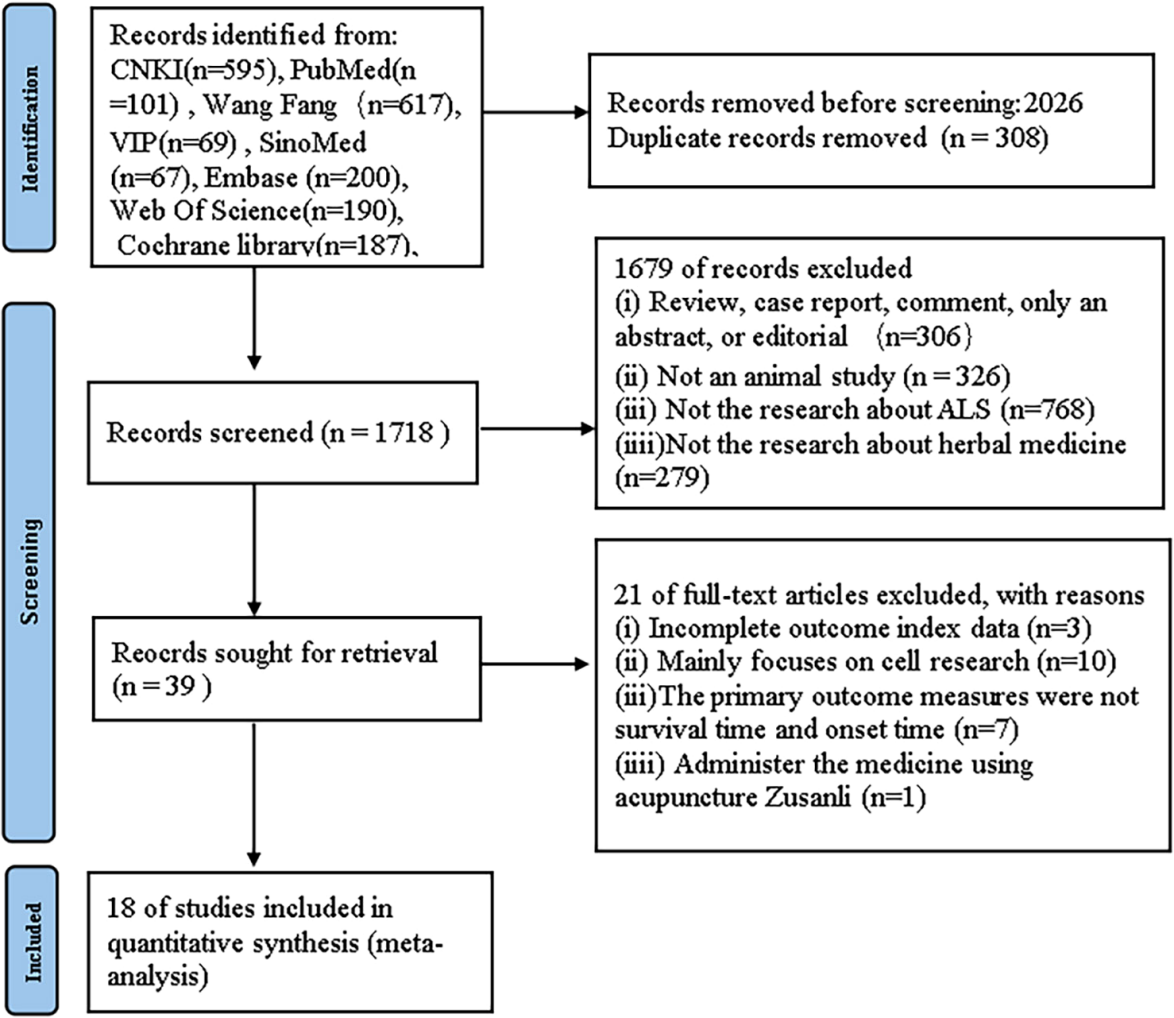
PRISMA diagram flaw.

For the preclinical evidence research section, we initially identified 2026 papers through systematic searches across six databases. After removing duplicates, 1718 records persisted. Upon careful examination of titles and abstracts, 1679 articles were excluded for one or more of the following reasons: (1) the article constituted a review, case report, comment, abstract-only, or editorial; (2) the article was not related to animal studies; (3) the article did not focus on research related to ALS; (4) the article did not focus on the therapeutic effects of HM. After thorough examination of the full text of the remaining 39 articles, 21 articles were excluded for one or more of the following reasons: (1) the primary outcome measures were not survival time and onset time, (2) incomplete outcome measure data, (3) primarily cell-based studies, and (4) intervention involving acupuncture at Zusanli (ST36) acupoint (**Figure 1**).

#### 3.1.2 Characteristics of included studies

We ultimately selected 18 studies (21–39) involving 19 comparisons, comprising 8 Chinese (22–28, 38) and 10 English (21, 29, 31–37, 39) publications. One study did two different comparisons because of the way they were treated (38). All 19 comparisons used SOD1^G93A^ mice as the experimental group, with each group exclusively receiving HM treatment. Fourteen comparisons (21–24, 26, 28, 29, 31, 33, 34, 36, 38, 39) included a blank control group, which was regularly administered saline or distilled water. Meanwhile, Six comparisons established a positive control group, five (24, 25, 32, 38, 39) of these used riluzole and one (38) used edaravone as the positive controls. Among these comparisons, seven utilized male animals exclusively (21, 22, 24, 32, 33, 36, 37), one utilized female animals exclusively (19), and three used an equal mix of males and females (25, 26, 28), eight comparisons did not specify the gender of the animals. Among the included comparisons, 6 employed a concentration gradient of HMs (26–28, 32, 38, 39). Nine comparisons administered treatments via oral gavage (22, 24, 26, 29, 32, 37–39), six via oral administration (21, 28, 29, 33, 34), two via intraperitoneal injection (36, 38) one via bilateral subcutaneous injection (33), and one did not specify the method of administration (25). Ten comparisons simultaneously recorded onset time and survival time as outcome measures (22, 23, 25–29, 32, 34, 39) For outcome measures, when a concentration gradient was employed, the highest concentration group was recorded (**Table 1**).

**Table 1:** Basic features of the included studies.

Study (year)	Animal model (age)	number(sex)		treatment			Route of administration Followup period	outcome index	Intergroup differences
		Trail (male/female)	Control (male/female)	intervention groups (mg/kg·d)	Positive control groups	Control			
Liu 2006 (28)	SOD1^G93A^ 70D	T1:18(8/10) T2:18(8/10)	C:19(9/10) WT:26(13/13)	T1: madecassoside (61.1 ± 11.0) T2: madecassoside (185.6 ± 18.7)	NO	C: Ordinary feed WT: Ordinary feed	Oral administration until death	1.onset time 2.Survival time 3.athletic ability	1 P>0.05 2.3 P<0.05
Cai 2019 (31)	SOD1^G93A^ 8W	T:24(UK/UK)	C:24(UK/UK) 24(UK/UK)	T2. Bojungikgi-tang 1000	NO	C: Ordinary feed WT: Ordinary feed	Oral administration 6W	1.Survival time 2.athletic ability	1 P<0.05 2. P<0.01
Kook 2017 (34)	SOD1^G93A^ 13W	T2:UK	C:12(0/12) WT:11(0/11)	T: KCHO-1 250	NO	C: NS WT: NS	Oral administration 4W	1.athletic ability 2.onset time 3.survival time	1.2.3 P<0.05
Cai and Yang 2019 (33)	SOD1^G93A^ 2M	T1:11(11/0) T2:11 (11/0)	C:8(8/0)	T1: Ordinary feed T2: Bojungikgi-Tang treated 1	NO	C: Ordinary feed WT : Ordinary feed	Oral administration 6W	1.athletic ability 2.survival time	1.2 P<0.05
Zhou 2016 (27)	SOD1^G93A^ 8W	T1:6(UK/UK) T2:6(UK/UK) T3:6(UK/UK)	6(UK/UK)	T1: Jianpi Yifei formula 28.6 T2: Jianpi Yifei formula 57.2 T3: Jianpi Yifei formula 114.4	NO	WT: NS	oral gavage until death	1.onset time 2.Survival time 3.athletic ability	1.2 P<0.01 3. P<0.05
Sujimoto 2021 (36)	SOD1^G93A^ 87D	T1:18(18/0) T2:18(18/0)	C1:18(18/0) C2:18(18/0)	T1: NS T2: Shenqi Fuzheng Injection 3	NO	C1: NS C2: Shenqi Fuzheng Injection 40ml/kg 3times a week	intraperitoneally injected until death	1.onset time	1. P<0.1
Shang 2021 (22)	SOD1^G93A^ 64D	T:19(19/0)	C:19(19/0) WT:19(19/0)	T: Bailing Capsule 0.01	NO	C: NS WT: NS	oral gavage 56D	1.Onset time 2.survival time	1. P>0.05 2. P<0.05
Xian 2018 (26)	SOD1^G93A^ 8W	T1:6(3/3) T2:6(3/3) T3:6(3/3)	C:6(3/3) WT:6(3/3)	T1: Jianpi Yifei formula 2.288 T2: Jianpi Yifei formula 1.144 T3: Jianpi Yifei formula 0.5522	NO	C: NS WT : NS	oral gavage until death	1.Onset time 2. delayed survival 2.athletic ability	1.2 P<0.01 3. P<0.05
Xu 2023 (23)	SOD1^G93A^ 90D	T1:16(0/16) T2:16(0/16)	C1:16(0/16) C2:16(0/16)	T1: NS T2: Lycium barbarum polysaccharide-glycoprotein 20	NO	C1: NS C2: Lycium barbarum polysaccharide-glycoprotein 20mg/kg·d	oral gavage 80D	1.Onset time 2.survival time	1. P>0.05 2. P<0.01
Winter 2018 (29)	SOD1^G93A^ 60D	T:9(UK/UK)	C:9(UK/UK) WT:9(UK/UK)	T: anthocyanin-enriched 2	NO	C: Ordinary feed WT: Ordinary feed	Oral administration until death	1. survival 2.Onset time 3.athletic ability	1. P<0.001 2.3. P<0.05
Yang 2022 (21)	SOD1^G93A^ 8W	T:8(8/0)	C:8(8/0) WT:8(8/0)	T: herbal formula extract 1	NO	C: distilled water WT:distilled water	Oral administration 6W	1.athletic ability 2.pathological manifestations	1.2 P<0.05
Dutta 2018 (37)	SOD1^G93A^ 50D	T1:25(UK/UK)	C2:25(UK/UK)	T1: Withania somnifera	NO	C: NS	oral gavage UK	1.athletic ability 2.survival time	1. P<0.01 2. P<0.05
Yang 2011 (35)	SOD1^G93A^ 98D	T1:11(11/0) T2:11(11/0)	C:10(10/0) C2:11(11/0)	T: Melittin	NO	C: NS	subcutaneously injected bilaterally 18D	1.Survival time 2.athletic ability	1. P>0.05 2. P<0.01
Li 2022 (38)	SOD1^G93A^ 12W(male) 13W (female)	T1:16 T2:17 T3:17 T4:18 T5:17	C1:12 C2:12	T1: NS T2: TBN 10 T3: TBN 30 T4: TBN 60	T5: Edaravone	C1: NS C2:TBN 30 mg/kg	intraperitoneal injection 4W	1.survival time 2.athletic ability	1. P>0.05 2. P<0.001
Li 2022 (38)	SOD1^G93A^ 6W	T1:17 T2:19 T3:20 T4:20	C:24	T1: NS T2: TBN 30	T3: Riluzole T4: Riluzole + TBN	C1: NS	oral gavage 14W	1.survival time	1. P<0.01
Yan 2022 (24)	SOD1^G93A^ 30D	T1:3*6(3*6/0) T2:3*7(3*7/0) T3:3*7(3*7/0)	3*7(3*7/0)	T1.NS T2.Jianpi Tongluo formula 630	T3. Riluzole	NS	oral gavage 30/70/110 D	1.athletic ability	1. P<0.05
Sekiya 2009 (39)	SOD1^G93A^ 6W	UK	UK	T1: Water T2: Wen-Pi-Tang 100 T3: Wen-Pi-Tang 200	T4: Riluzole	C1: Water	oral gavage UK	1. survival time 2.Onset time 3.athletic ability	1. P>0.05 2.3 P<0.05
Zhu 2017 (25)	SOD1^G93A^	T1:8(3/5) T2:8(4/4)	C8(3/5)	T1: Flavored Sijunzi decoction	T2: Riluzole	C: NS	UK UK	1.onset time 2. delayed survival 2.athletic ability	1.2 P<0.001 2. P<0.05
Zhou 2018 (32)	SOD1^G93A^ 57D	T1:9(9/0) T2:9(9/0) T3:9(9/0) T4:9(9/0) T5:9(9/0) T6:9(9/0)	C:9(9/0)	T1: HLSJ 30 T2: HLSJ 45 T3: HLSJ 60 T4: HLSJ 75 T5: Glucose	T6: Riluzole	C: double distilled water	oral gavage until death	1. survival time 2.Onset time	1. P>0.05 2. P<0.01

T, Experimental group; T1;2…, Specific group; C, Control group; C1;2…, Specific group; WT, Wild type; UK, Unknown; TBN, ligustrazine derivatives HLSJ, Huolingshengji Formula; NS, Normal saline.

#### 3.1.3 Study quality and risk of bias

##### 3.1.3.1 CAMARADES

The quality scores of the studies ranged from 4 to 8, with a total score of 10. One study received a score of 4 (25); two studies received a score of 5 (29, 38); four studies received a score of 6 (23, 24, 37, 39); seven studies received a score of 7 (23–25, 28, 29, 32, 33), and four studies received a score of 8 (21, 22, 33, 34). All included records were peer-reviewed publications, and all studies utilized appropriate animal models without the use of anesthetics with marked intrinsic properties. Twelve studies mentioned random allocation of animals into treatment and control groups (21–23, 25–28, 31–33, 36, 39), the methods mentioned in the two studies for specific randomization included random number table and sequential numbering (26, 29). three studies reported blinded outcome assessment (22, 33, 34), however, no studies reported sample size calculations. Twelve studies described temperature control (21, 22, 24, 26–28, 38, 39), twelve studies reported compliance with animal welfare regulations (22, 24, 26–28, 31, 33–37, 39), and seven studies declared no potential conflicts of interest (21, 29, 32–36) (**Table 2**).

**Table 2:** Quality assessment of included studies.

Studys	A	B	C	D	E	F	G	H	I	J	TOTAL
liu 2006 (28)	√	√	√	√	×	√	√	×	√	×	7
Zhou 2016 (27)	√	√	√	√	×	√	√	×	√	×	7
Cai 2019 (31)	√	√	√	√	×	√	√	×	√	×	7
Kook 2017 (34)	√	√	√	√	√	√	√	×	√	√	8
Sujimoto 2021 (36)	√	×	√	√	√	√	√	×	√	×	7
Li 2022 (38)	√	√	×	√	×	√	√	×	×	×	5
Shang 2021 (22)	√	√	√	√	√	√	√	×	√	×	8
Xian 2018 (26)	√	√	√	√	×	√	√	×	√	×	7
Xu 2023 (23)	√	×	√	√	√	√	√	×	×	×	6
Cai and Yang 2019 (33)	√	√	√	√	×	√	√	×	√	√	8
Winter 2018 (29)	√	×	×	√	×	√	√	×	×	√	5
Sekiya 2009 (39)	√	√	×	√	×	√	√	×	√	×	6
Yang 2011 (35)	√	√	×	√	×	√	√	×	√	√	7
Zhu2017 (25)	√	×	×	√	×	√	√	×	×	×	4
Dutta 2018 (37)	√	×	√	√	×	√	√	×	√	√	6
Zhou 2018 (32)	√	√	√	√	×	√	√	×	×	√	7
Yan 2022 (24)	√	√	×	√	×	√	√	×	√	×	6
Yang 2022 (21)	√	√	√	√	×	√	√	×	√	√	8

A. Publication in a peer-reviewed journal; B.Statement of temperature control; C. Randomization to treatment group; D. Allocation concealment; E. Blinded assessment of outcome; F. Avoidance of anesthetics with known notable intrinsic neuroprotective properties; G. Use appropriate ALS animal models; H. Sample size calculation; I. Compliance with animal welfare regulations; J. declared any potential conflict of interest.

##### 3.1.3.2 SYRCLE

The SYRCLE’s ROB is currently the only tool specifically designed for evaluating the internal validity of animal experiments. The risk of bias scores of the studies ranged from 3 to 7, with a total score of 10. Two studies received a score of 3 (38, 39); Five studies received a score of 4 (24, 25, 34, 35, 37); Seven studies received a score of 5 (23, 26, 29, 31, 33); Three studies received a score of 6 (22, 28, 35), One studies received a score of 7 (36). It is developed based on the Cochrane Bias Risk Assessment Tool and are additional items. As shown in **Table 3**, within the 10 items: A. Sequence generation: two studies used the “random number table method” for grouping, rated as “low risk”; Ten studies only mentioned “random” without detailed explanation, and six studies did not mention the grouping method, rated as “uncertain risk” (the quality assessment table still needs modification). B. Baseline characteristics: three studies mentioned baseline comparison of mice, rated as “low risk.” The remaining fifteen studies mentioned only one or more of the age, gender, weight, or species of rats, and did not provide baseline values of relevant outcome indicators in the experiment, hence rated as “uncertain risk.” C. Allocation concealment: two studies mentioned “random” or “random number table,” rated as “low risk”; the remaining sixteen studies did not mention concealment of allocation or the provided information was insufficient to achieve the unpredictability of the random sequence, hence rated as “uncertain risk.” D. Random housing: fifteen studies indicated placing mice in individually housed environments with free access to water, similar temperature, humidity, etc., rated as “low risk.” 3 studies did not mention housing conditions, rated as “uncertain risk.” E. Performance bias (Blinding): All studies did not describe blinding of animal caregivers, researchers, and outcome assessors, hence rated as “uncertain risk.” F. Outcome assessment: three studies mentioned “random” selection of mice for outcome assessment, rated as “low risk”; fifteen studies did not mention it, hence rated as “uncertain risk.” G. Detection bias (Blinding):one study mentioned blinding in evaluating experimental results, rated as “low risk”; seventeen studies did not mention it, hence all rated as “uncertain risk.” H. Incomplete outcome data: one study had missing data during the experiment, but did not provide any explanation on whether the missing data affected the final result’s authenticity, hence rated as “high risk”; four studies only reported the data range, making it impossible to determine if there was data missing, hence rated as “uncertain risk.” I. Selective outcome reporting: All studies did not find incomplete data reporting, rated as “low risk.” J. Other sources of bias: All studies did not find other sources of bias, hence rated as “low risk.” (**Supplementary Data Sheet 3**).

**Table 3:** Basic features of the included studies.

Author	Year	Journal	Nation	Experimental method	Involved mechanism	Involving berbs
Ding Huang,Tang San et al. (40)	2019	Chinese Pharmacology Bulletin	China	Animal experiment	1	Borneol,Astragaloside, Total notoginseng
Xu Lu,Zhang Taijun et al. (41)	2016	Information Journal of Chinese Medicine	China	Cell experiment	1	Borneol
Wang Jian,Qu Xiaolan et al. (42)	2018	Chinese Journal of Hospital Pharmacy	China	Animal experiment	1	Borneol,safflower
Tang Bingrong,Li Hua et al. (43)	2019	Journal of Chinese Experimental Formulae	China	Animal experiment	1	Sijunzi Decoction
Rao Xiao,Tang Tibo et al. (44)	2014	Information Journal of Chinese Medicine	China	Animal experiment	1	Buyang Huanwu Decoction
Liu Yangbo,Chen Haodong et al. (45)	2023	Chinese Journal of Pharmacy	China	Animal experiment	1	Ginsenoside Rg1
Zhong Xiaoqin,Luo Chuanjin et al. (46)	2019	Experimental and Therapeutic Medicine	China	Cell experiment	1	Breviscapine
Zhang Qiuyan,Wang Lingling et al. (47)	2011	Journal of Intractable Diseases	China	Animal experiment	1	Jiweiling lyophilized powder
Li Yinghui,Liu Shaobo et al. (48)	2017	Acta Physiologica Sinica	China	Cell experiment	2	Tripterygium wilfordis
Yang Eun Jin,Kim Seon Hwy et al. (35)	2011	Journal of Neuroinflammation	Korea	Animal experiment	2	Bee venom,Melittin
Lee Kang-Woo,Ji Hye Min et al. (49)	2013	Journal of Ethnopharmacology	Korea	Cell experiment	2	Human placenta extract
Wu Jie,Wang Bin et al. (50)	2019	European Journal of Pharmacology	China	Cell experiment	2	Gardenia,Geniposide (GEN)
Shi Ruili,Qi Ruifang et al. (51)	2017	New Chinese medicine and clinical pharmacology	China	Cell experiment	2	Melon seed gold
Kook Myung Geun,Choi Soon Won et al. (34)	2017	Journal of Veterinary Science	Korea	Animal experiment	2	Turmeric,Danshen root, Tall gastrodia tuber, Common floweringqince fruit
Hsu Chien-Chin,Kuo Ting-Wei et al. (52)	2020	Journal of Neuroimmune Pharmacology	China	Animal experiment	2	Astragaloside IV, Total saponins of Japanese ginseng, Wogonin
Zhang Xuejun,Yi Tingjin et al. (53)	2011	Research and development of natural products	China	Pharmacological experiment	3	Polysaccharide component
Zeng Min,Xu Huifang et al. (54)	2018	Modern Chinese medicine research and practice	China	Pharmacological experiment	3	Aucklandia
Ruan Shunan,Lu Yan et al. (55)	2013	Chinese journal of traditional Chinese Medicine	China	Pharmacological experiment	3	Cablin patchouli herb
Shen Lulu,Lu Yan et al. (56)	2013	Chinese Traditional and Herbal Drugs	China	Pharmacological experiment	3	Giant knotweed rhizome
Du Dongsheng,Cheng Zhihong et al. (57)	2017	Chinese journal of traditional Chinese Medicine	China	Pharmacological experiment	3	Tokyo violet herb
Jin Jiahong,Cheng Zhihong et al. (58)	2012	Journal of Chinese Pharmaceutical Sciences	China	Pharmacological experiment	3	Dayflower herb
Yazdani Solmaz, Seitz Christina et al. (59)	2022	Nature Communications	Sweden	Clinical trials	4	No
Ding Xuping,Wang Xue et al. (60)	2021	World Science and technology - TCM modernization	China	Cell experiment	4	Trichosanthes
Murdock Benjamin J,Zhou Tingting et al. (61)	2017	JAMA Neurology	America	Clinical trials	5	NO
Garofalo Stefano,Cocozza Germana et al. (62)	2020	Nature Communications	Italy	Clinical trials and Animal experiment	5	NO
Li Jianming,Peng Lishan et al. (63)	2022	Journal of Shenyang Pharmaceutical University	China	Animal experiment	5	Futenge mixture,Futenge, milkvetch root,red ginseng
Cheng Qi,Li Ning et al. (64)	2013	Journal of Ethnopharmacology	China	Animal experiment	5	Fuzheng huayu
Jin Mei Ling,Park Sun Young et al. (65)	2013	Environmental Toxicology and Pharmacology	Korea	Cell experiment	6	Acanthopanax extract
Mahesh Ramalingam,Jung Hyo Won et al. (66)	2012	Phytotherapy research: PTR	Korea	Cell experiment	6	Cryptotanshinone
Sun Meng-Meng, Bu Hui et al. (67)	2009	Neurological Research	China	Pharmacological experiment	7	Allicin
Kanekura Kohsuke,Hashimoto Yuichi et al. (68)	2005	Journal of Biological Chemistry	Japan	Pharmacological experiment	6	Cryptotanshinone
Guo Yansu,Zhang Kunxi et al. (69)	2011	Brain Research	China	Animal experiment	7	Allicin
Li Shu-Yan,Jia Yu-Hong et al. (70)	2010	Free Radical Biology and Medicine	China	Animal experiment	8	Chuan Xiongqin
Callewaere Céline,Banisadr Ghazal et al. (71)	2006	Proceedings of the National Academy of Sciences of the United States of America	France	Pharmacological experiment	8	Chuan Xiongqin
Chen Zhao,Pan Xueke et al. (72)	2013	Cancer Letters	China	Cell experiment	8	Chuan Xiongqin

The numbers in the Related mechanism column indicate the following mechanisms 1.Blood-brain barrier protection 2.Anti-neuroinflammation 3.Inhibition of complement system activation 4.Regulate T lymphocytes 5.Regulates natural killer cells 6.Excitatory amino acids toxicity 7.Oxidative stress 8.Cytotoxicity of calcium.

#### 3.1.4 Effectiveness

##### 3.1.4.1 Onset time

###### 3.1.4.1.1 Meta analysis

**Figure 2:**
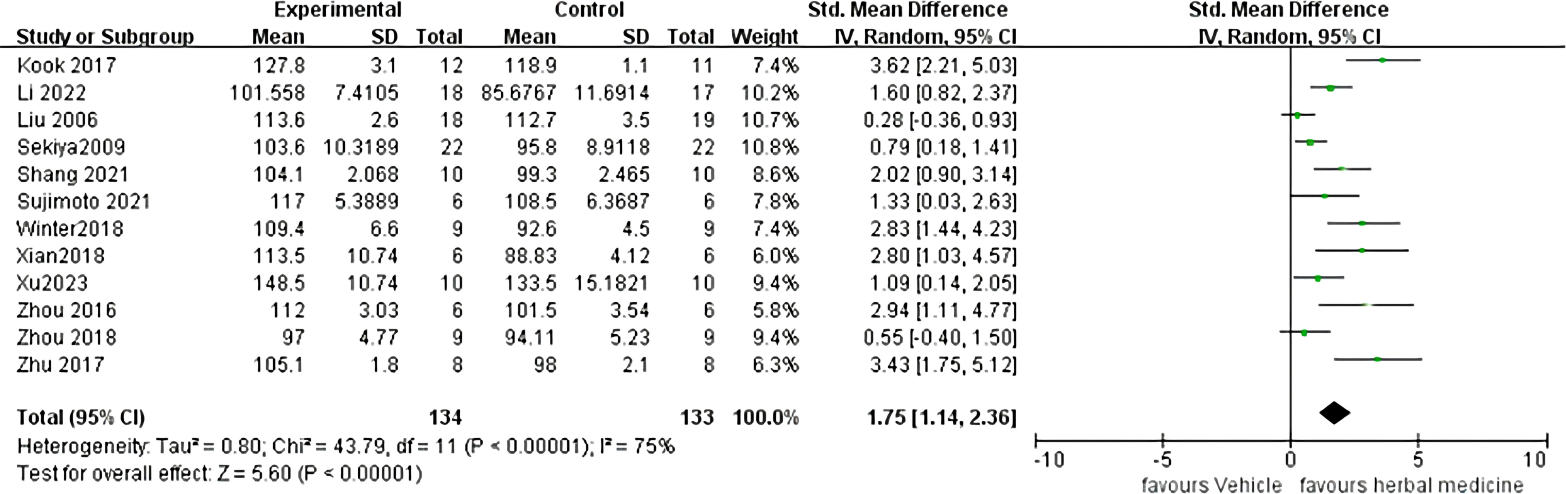
ALS onset time Meta analysis.

Twelve studies were included (22, 23, 25–29, 34, 36, 38, 39), with a total sample size of 267animals, including 134 animals treated with HM and 133 animals in the control group, with individual study sample sizes ranging from 5 to 25. Heterogeneity test results showed *I²*= 75%, P < 0. 01. A random-effects model was used. The overall effective rate Standardized Mean Difference(SMD) was 1.75, 95% Confidence Interval(CI) (1.14 ~ 2.36), Z = 5.60, P < 0.01, indicating that HM treatment was effective and superior to the control group (P < 0.01) (**Figure 2**).

###### 3.1.4.1.2 Sensitivity analysis

**Figure 3:**
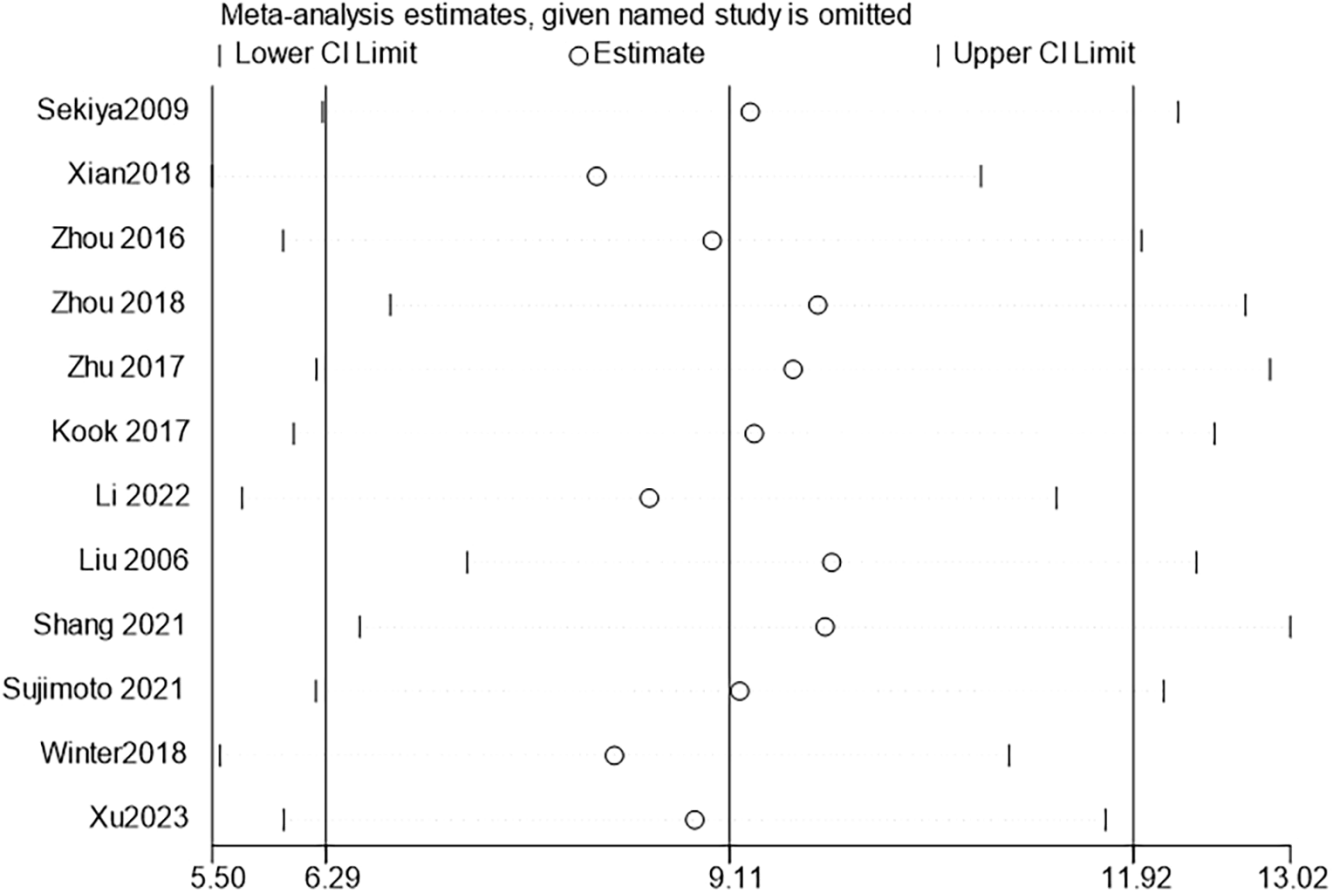
ALS onset time sensitivity analysis.

Further sensitivity analyses comparing HM with conventional feeding regimens (12 trials (22, 23, 25–29, 32, 34, 36, 38, 39) with 267 participants) showed that Chinese HM was more beneficial in terms of overall mean reduction in onset time, with no significant heterogeneity between studies (**Figure 3**).

##### 3.1.4.2 Survival time

###### 3.1.4.2.1 Meta analysis

**Figure 4:**
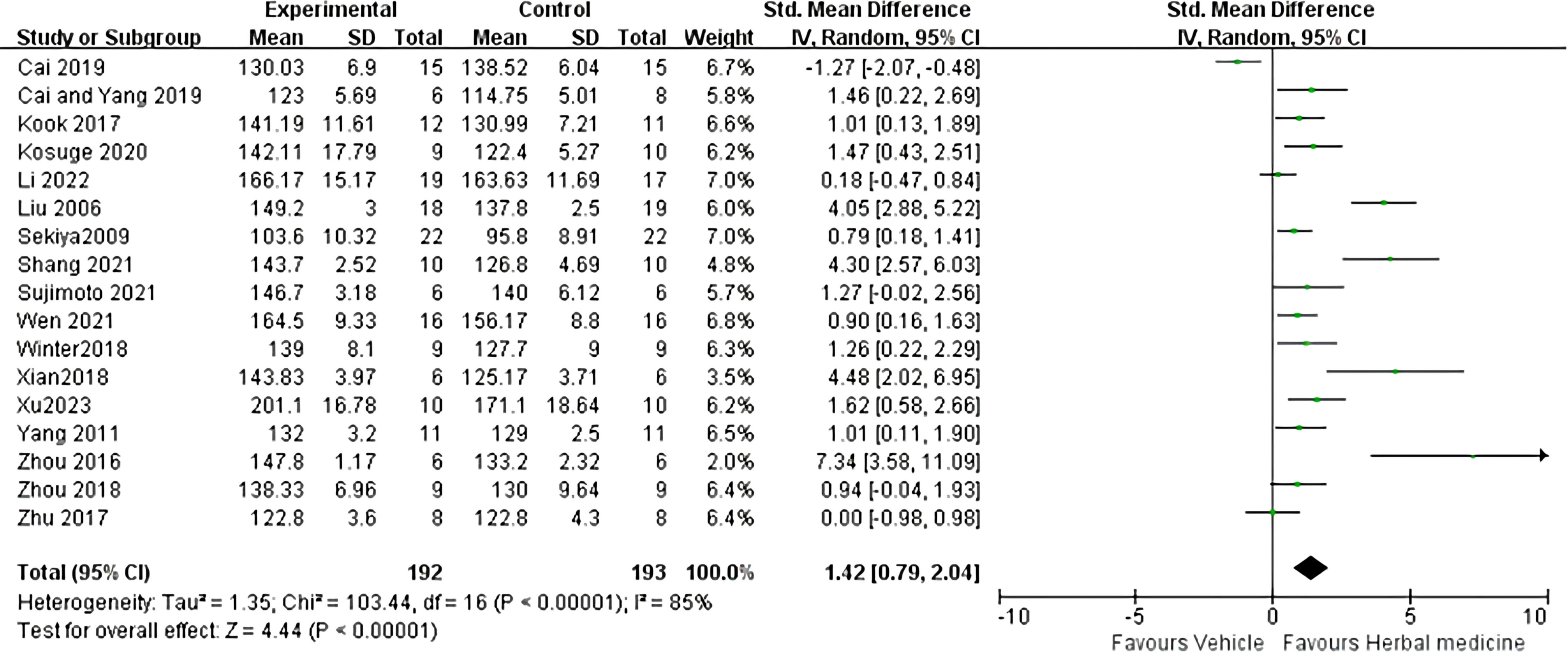
ALS survival time Meta analysis.

Seventeen studies were included (22–29, 31–39), with a total sample size of 385 animals, including 192 animals treated with HM and 193 animals in the control group, with individual study sample sizes ranging from 5 to 25. Heterogeneity test results showed *I²* = 85%, *P* < 0.01. A random-effects model was used. The overall effective rate SMD was 1.42, 95% CI (0.79 ~ 2.04), Z = 4.44, *P* < 0.01, indicating that HM treatment was effective and superior to the control group (*P* < 0.01) (**Figure 4**).

###### 3.1.4.2.2 Sensitivity analysis

**Figure 5:**
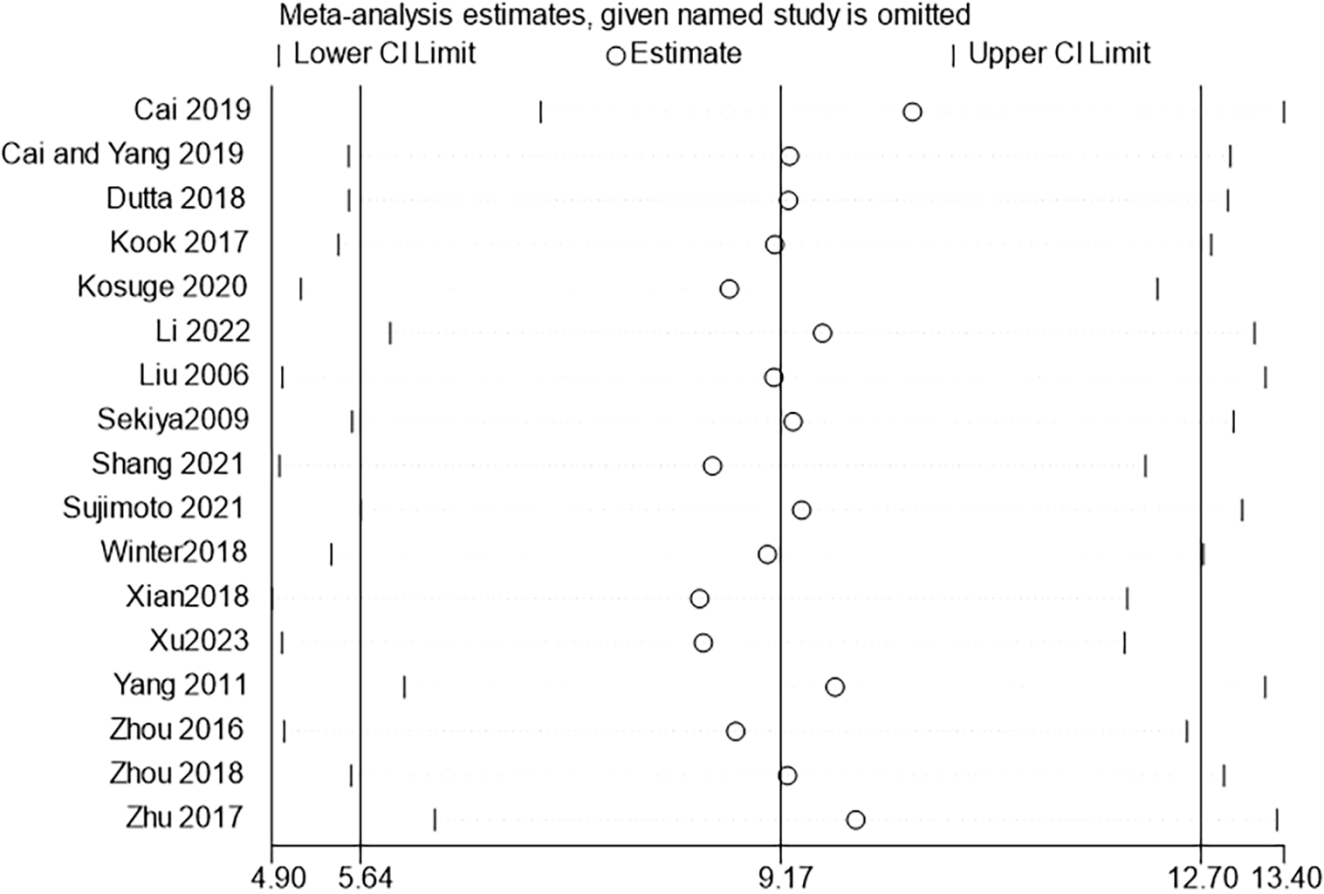
ALS onset time sensitivity analysis.

Further sensitivity analyses comparing HM with conventional feeding regimens (17 trials (22–29, 31–39) with 385animals) showed that HM was more beneficial in terms of overall mean reduction in survival time, with no significant heterogeneity between studies (**Figure 5**).

##### 3.1.4.3 Stride length

**Figure 6:**
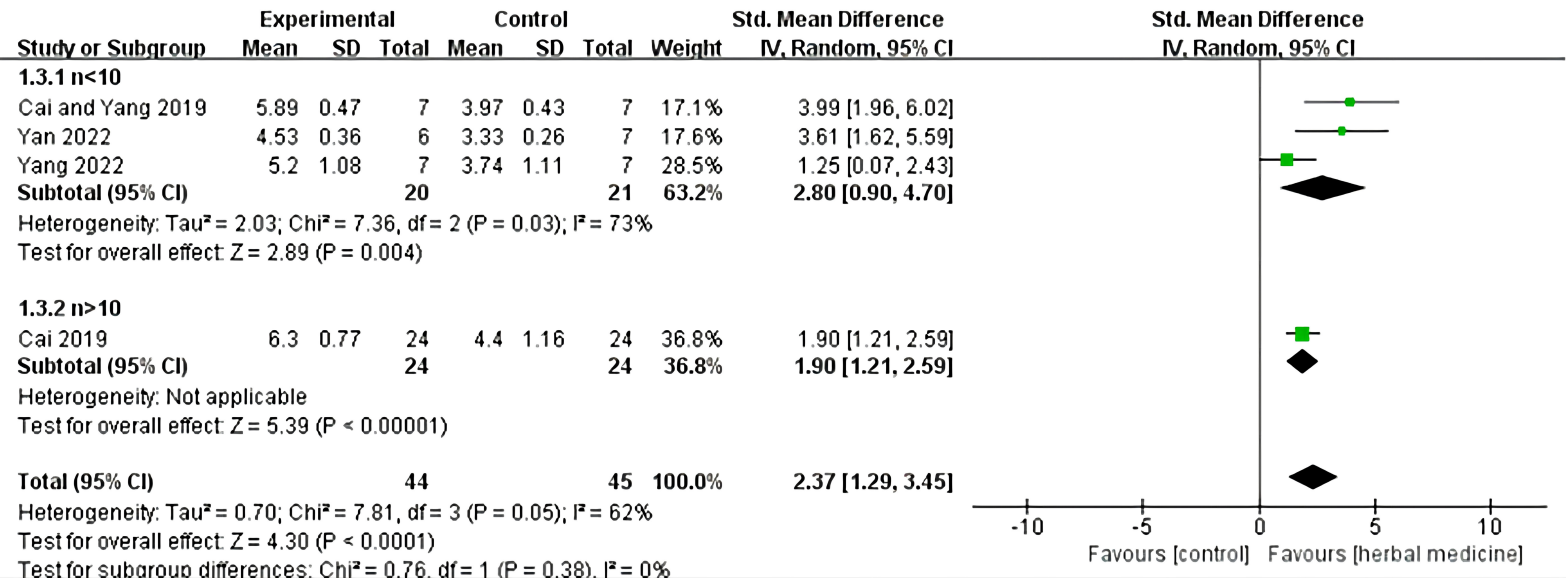
ALS stride length Meta analysis.

Four studies were included (20, 23, 30, 32), with a total sample size of 89 animals, including 44 animals treated with HM and 45 animals in the control group, with individual study sample sizes ranging from 5 to 25. Heterogeneity test results showed *I*²= 62%, *P* = 0.05. A random-effects model was used, and further subgroup analysis was conducted, dividing the samples into two groups based on sample size: n > 10 and n < 10. In the n < 10 group, three studies were included, with a total sample size of 41 animals, including 20 animals treated with HM and 21 animals in the control group. The overall effective rate SMD was 2.80, 95% CI (0.90 to 4.70), Z = 2.89, *P* = 0.004, indicating that HM was effective in treating ALS in the n < 10 group and its efficacy was superior to the control group (*P* < 0.05). In the n > 10 group, one study was included (18), with a total sample size of 48 animals, including 24 animals treated with HM and 24 animals in the control group. The overall effective rate SMD was 1.90, 95% CI (1.21 to 2.59), Z = 5.39, *P* < 0.01, indicating that HM was effective in treating motor neuron diseases compared to the control group in the n > 10 group (*P* < 0.01) (**Figure 6**).

##### 3.1.4.4 Duration time

**Figure 7:**
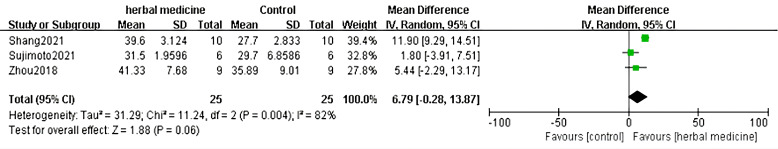
ALS duration time Meta analysis.

In the 18 studies included, 3 studies (22, 32, 36) assessed the therapeutic effect by calculating the duration of disease in ALS mice, with records of disease duration in both the treatment and control groups. Meta-analysis was conducted on the durations of the two groups to evaluate the efficacy of HM in treating ALS animal models. In the 3 studies, both the treatment and control groups included 25 animals. The heterogeneity test showed *P* =0.004, *I* ^2^ = 82%, indicating statistical significance in heterogeneity between groups. Therefore, a random-effects model was used to combine the effect sizes of disease duration. The results showed that the disease duration in the treatment group was shorter than that in the control group (MD=6.79, 95% CI [-0.28, 13.87]), but the difference between the two groups was not statistically significant (*P* =0.06). Regarding the treatment of ALS mice, using recorded disease duration for efficacy evaluation suggests that HM has no effect in delaying the progression of ALS compared to the control group (**Figure 7**).

### 3.2 Scoping review

#### 3.2.1 Study inclusion

**Figure 8:**
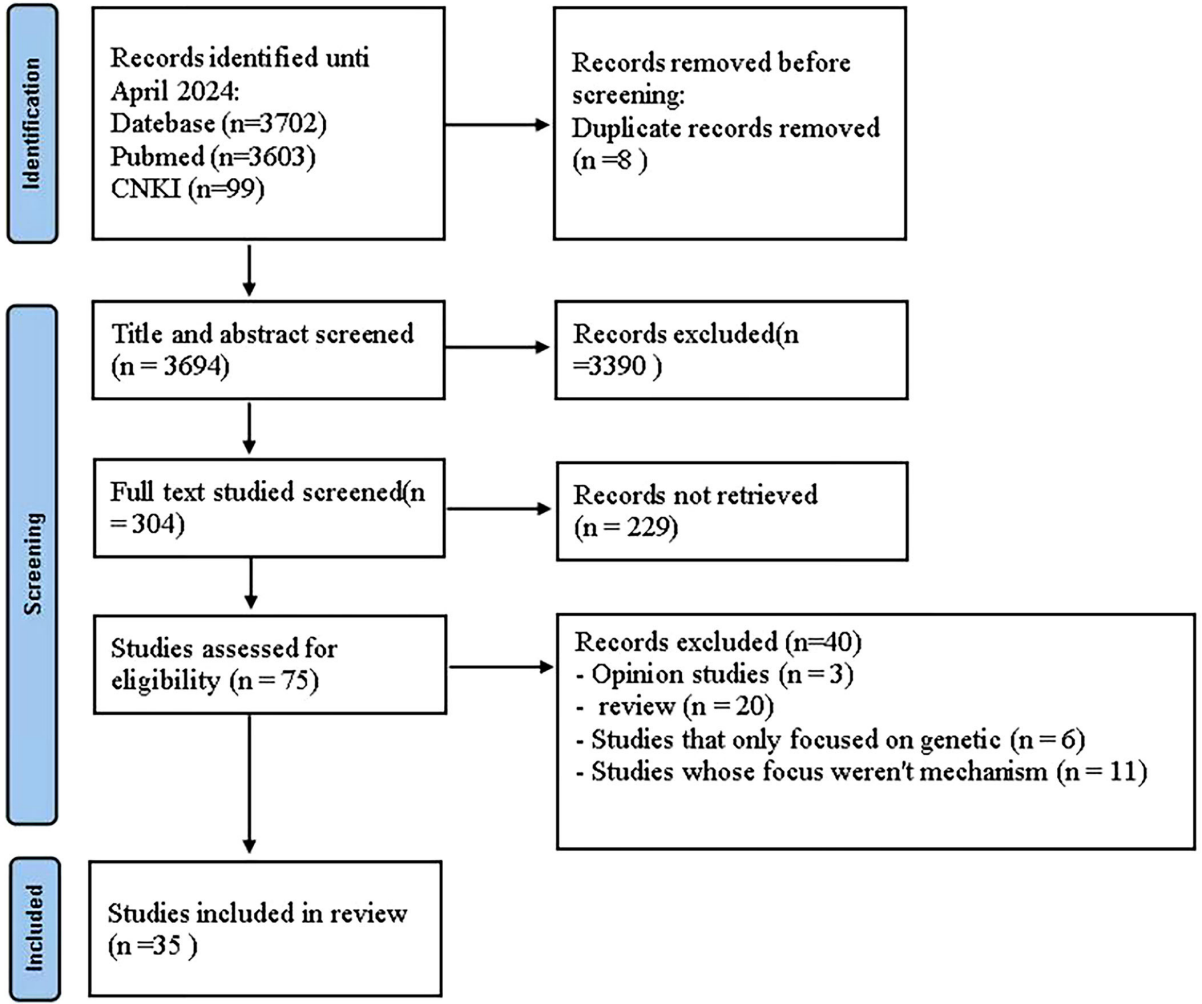
PRISMA diagram flaw.

For the scoping review section on the immune mechanisms of HM in the treatment of ALS, a total of 3702 articles were extracted from the initial search carried out in two databases. After removing the duplicates, 3694 articles were selected for the first analysis by title and abstract. The full-text analysis included 75 articles, of which 35 were considered for this scoping review (**Figure 8**).

#### 3.2.2 Characteristics of included studies

This scoping review encompasses a total of 35 studies, with 25 from China, 5 from Korea, 1 from Japan, and 4 from other Western countries. The included studies range from as early as 2005 to as recent as 2023. There are 13 studies on animal experiments, 10 on cell experiments, 3 on clinical trials, and 9 on pharmacological experiments. Five immune modulation pathways are covered, along with three other mechanisms. The literature on blood-brain barrier protection is the most abundant, with 8 studies. In total, 26 different single drugs and compound formulations are involved (**Table 3**).

#### 3.2.3 Mechanisms of HM in the treatment of ALS

ALS is an incurable neurodegenerative disease that affects the upper and lower motor neurons of the spinal cord, the cerebral cortex, and the spinal cord. The etiology and pathogenesis of ALS remain unknown at present (1). Here, some studies indicates that HMs show promising potential in combating oxidative stress, excitatory amino acid toxicity, nerve inflammation, and calcium cytotoxicity, offering hope in the treatment of ALS.

##### 3.2.3.1 Excitatory amino acids toxicity

In 1957, Lucas and Newhouse’s (73) pioneering research demonstrated the lethal effects of glutamate on neurons in the central nervous system (CNS).Following this, The molecular mechanisms underlying neuronal injury due to excessive glutamate receptor stimulation are starting to be unraveled, indicating that glutamate may exert toxicity on neurons through multiple pathways (74). The excitotoxicity resulting from the abnormal elevation of extracellular excitatory neurotransmitter glutamate, including the generation of free radicals and lipid superoxide, can induce spontaneous dissolution and degeneration of neurons, contributing to the development of ALS. Numerous experimental findings demonstrate the ability of cryptotanshinone to counter glutamate-induced cytotoxicity and safeguard neurons, indicating its potential utility in mitigating the onset of ALS. The pivotal involvement of the PI3K/Akt signaling pathway in cell survival against glutamate-induced toxicity has been underscored (68). several Chinese herbs exhibit the capability to inhibit amino acid toxicity and consequently shield neurons. For instance, Acanthopanax extract elevates heme oxygenase (HO)-1 expression, thereby curbing the generation of NO/ROS induced by LPS. Notably, HO-1 expression serves to safeguard cells against glutamate-induced neuronal demise (65). Additionally, Cryptotanshinone-mediated neuroprotection combats glutamate-induced toxicity by activating the PI3K/Akt pathway and averting the downregulation of Bcl-2 within the anti-apoptotic protein family. Furthermore, Mahesh’s research revealed cryptotanshinone’s capacity to hinder nerve cell apoptosis induced by sodium nitroprusside (SNP), thus exhibiting neuroprotective properties (66).

##### 3.2.3.2 Oxidative stress

Oxidative stress is caused by an imbalance in the production and removal of Reactive oxygen species (ROS) and Reactive nitrogen species (RNS) (75). Oxidative stress can cause oxidative modification of bioactive molecules such as proteins, lipids, sugars, nucleic acids, etc., so that they lose their original structure and function, affect the normal physiological function of cells, and finally lead to cell degeneration and necrosis. Under normal conditions, free radicals do not cause pathological changes in the body, because the body has enzymes to fight free group damage, such as SOD1, glutathione peroxidase (GSH), catalase (CAT) and non-enzymatic system defense system. Such as non-enzymatic antioxidants carotenoids, tocopherols and vitamin C, as well as free metals and heme-binding proteins. They can suspend the free radical chain reaction, or turn the free radical into a less active substance, so that the production and removal of free radicals are in balance. If the production of free radicals exceeds the body’s ability to remove them, the body will experience oxidative stress (76). Allicin, the primary compound in garlic oil, has demonstrated its ability to induce phase II enzymes, thereby enhancing antioxidant activity and protecting ALS neurons from oxidative stress (67). Administering allicin orally with DATS to SOD1-G93A mice at clinical onset induced the expression of HO-1 in the lumbar spinal cord, directly influencing oxidative damage. These findings suggest that oral administration of DATS significantly extends the lifespan of mice (69).

##### 3.2.3.3 Cytotoxicity of calcium

In classical acute excitatory toxicity, the influx of Na+ and Cl- disrupts intracellular Ca2+ homeostasis, triggering a cascade of detrimental biochemical processes. Opening of voltage-gated calcium channels leads to a surge in calcium ions, resulting in excessive release of the excitatory amino acid glutamic acid. This influx of calcium ions through NMDA/AMPA receptors, metabolic glutamic acid receptors, and voltage-dependent calcium channels activates enzymes such as proteases, lipases, kinases, nucleases, and NOS. The generation of free radicals and synthesis of NO further exacerbate neuronal damage, ultimately leading to programmed cell death via apoptosis gene activation. Neuroprotective drugs are thought to primarily act by preventing calcium influx, regulating excitatory amino acid toxicity, and modulating microvascular inflammatory responses. Studies have shown that an extract from the Chinese herb Chuan Xiongqin, can protect nerve cells by lowering intracellular calcium levels and inhibiting glutamate release (70). Callewaere et al. (71) investigated the protective effect of ligustrazine by stimulating nerve cells with stromal cell-derived factor (SDF-1), which elevates intracellular calcium levels, and then treating them with ligustrazine. They observed a significant decrease in intracellular calcium levels in the ligustrazine-treated group exposed to SDF-1, indicating ligustrazine’s ability to mitigate calcium cytotoxicity and serve as a neuroprotective agent (72).

##### 3.2.3.4 Other relevant mechanisms

Some studies have also found that the incidence of ALS is related to neurotrophic factor deficiency, metal and trace element imbalance, cell apoptosis, viral infection and abnormal neurofilament aggregation.

#### 3.2.4 The possible immunological mechanisms of HM in treating ALS

HM has better anti-inflammatory properties and extensive immunomodulatory effects, and can have better curative effect in the diseases of immune system disorders. ALS can be alleviated and treated by protecting the blood-brain barrier, anti-neuroinflammation, inhibiting the activation of complement system, inhibiting natural killer cytotoxicity and regulating T cells (77).

##### 3.2.4.1 Blood-brain barrier protection

Blood central nervous system barrier includes blood-brain barrier and blood-spinal barrier, which can control the transport of substances on both sides of the barrier, thus protecting the relatively stable environment in nervous tissue. It is an important immune barrier for ALS, which can effectively prevent toxic substances from infiltrating into the central nervous system from the blood and pump toxins outward (78). The target of HM protecting blood brain barrier is closely related to tight junction protein. Tight junction protein is composed of three peripheral cytoplasmic proteins, namely, occludingprotein, closure protein and attachment molecule, and closed small cyclic proteins (ZO-1, ZO-2 and ZO-3).Both borneol and astragaloside can increase the expression of atretic zonule 1 and closure protein 5, and their combination with total notoginseng can also inhibit the downregulation of atretic zonule 1 and occlusion protein, thus significantly improving the permeability of the blood-brain barrier (40). If borneol was administered with safflower, the expression of MMP-2 and MMP-9 could be decreased, and the expression of ZO-1 and closure protein 5 could be increased (42). Sijunzi Decoction can increase the expression levels of occlusal protein, ZO-1, closure protein 1 and their mRNA (43). The expression of von Willeophilia factor in serum, vascular endothelial growth factor, MMP-9 and MMP-2 in brain tissue decreased after the use of Buyang Huanwu Decoction, indicating the protective effect of the Blood-brain barrier (44). Ginsenoside Rg1 can also up-regulate the levels of atretic zonolin-1 and occlusion-protein, and down-regulate the expression of matrix metalloproteinase-2 and matrix metalloproteinase-9 to restore the integrity of the Blood-brain barrier (45). Breviscapine mainly plays its role by up-regulating the expression of CD63 and the blood-brain barrier tight junction proteins claudin5, occludin and ZO-1 (46). Jiweiling related preparations such as Jiweiling lyophilized powder can significantly improve blood-brain barrier score, delay neuronal edema and reduce blood-brain barrier permeability in damaged mice (47). By regulating the permeability of the blood-brain barrier, the active components of Chinese medicine can restore the integrity of the immune barrier in ALS and enhance its self-protection.

##### 3.2.4.2 Regulates microglia

Neuroinflammation is an important host defense mechanism that protects the brain from infection or injury and restores normal structure and function (79, 80), chronic inflammation can induce cytotoxicity and worsen the severity of different neurodegenerative diseases, such as Parkinson’s disease (PD) (81), multiple sclerosis (MS) (82), and ALS (83). Dysregulation of the inflammatory response, characterized by abnormal activation of microglia and overabundance of pro-inflammatory cytokines, leads to the neurodegeneration observed in ALS (84). Chinese HM has rich historical background, remarkable curative effect and minimal adverse reactions. It acts by regulating microglia activation and polarization, inhibiting inflammatory responses, and by mediating microglia and various related pathways such as NF-κB signaling pathway, Toll-like signaling pathway, Notch signaling pathway, AMPK signaling pathway, MAPK signaling pathway, MAPK signaling pathway, etc (85).Tripterygium wilfordis extract (Tripterygium wilfordis) can regulate the phosphorylation of kinase 1/2 and nuclear factor kB by blocking extracellular signals to reduce the production of pro-inflammatory factors and nitric oxide, so as to inhibit the anti-inflammatory effect of autoimmune (86). As resident macrophages of the central nervous system, microglia play a key role in maintaining brain homeostasis (87), but if microglia are over-activated, it will lead to the release of many pro-inflammatory factors and neurotoxic substances, aggravating the damage. Melittin, an effective component extracted from bee venom, can directly reduce the activity of microglia or indirectly reduce the secretion of inflammatory factors and the phosphorylation level of P38 mitogen-activated protein kinase in brain stem and spinal cord, significantly regulate the inflammation of ALS mice and delay the development of the disease (35). The extract can inhibit C-JUN amino-terminal kinase signaling pathway, reduce the expression of microglia-induced nitric oxide synthase and cyclooxygenase-2, and play an anti-inflammatory role (49). The up-regulated expression of TLR4 is a key receptor involved in the activation and function of microglia. The active ingredient Geniposide (GEN) in Gardenia can effectively reduce the expression of TLR4, MyD88, p-IκB, NF-κB, p-ERK1/2 and p38 proteins, thereby playing an anti-inflammatory role and inhibiting the activation of microglia by down-regulating the TLR4-dependent pathway of MyD88 (50). The triterpenoid saponin compound polyhemigosaponin F (PGSF) extracted from melon seed gold of Polygala can effectively counteract the up-regulation of toll-like receptor 4 (TLR4) in microglia, and down-regulate the expression of nitric oxide synthase (iNOS) and circular cell enzymase-2 (COX-2) induced by cellular inflammatory proteases. Improve the overactivation of microglia and the production of neurotoxic factors, and reduce the damage of nerve cells (35). KCHO-1, a natural ethanol extract obtained from turmeric, salvia miltiorrhizae, Tianma, papaya and other herbs, can reduce oxidative stress by decreasing gp91phox subtype expression of NADPH oxidase, down-regulating the level of induced-nitro oxide synthase, and alleviating the phosphorylation of P38 mitogen-activated protein kinase and the activation of extracellular signal-regulated kinase 1/2. Inhibit microglia proliferation and activation (34). Astragaloside IV, total saponins and baicalin can regulate microglia polarization and improve brain tissue inflammation by mediating MAPK signaling pathway. Calycosin has the ability to reduce TNF-α-containing microglia populations by activating the BDNF/TrkB signaling pathway, thereby reducing inflammation and neuronal damage (52).

##### 3.2.4.3 Inhibition of complement system activation

The complement system is a group of activated enzymatically active proteins in human serum and interstitial fluid. It is composed of more than 30 kinds of soluble proteins and membrane binding proteins synthesized by the liver. The complement consists of nine components, named C1, C2, C3,… and C9 (88). Normal activation of complement is beneficial to enhance immunity, but excessive activation can cause inflammation, tissue damage and various immune hemolysis reactions (89). Complement activation has long been implicated in the pathogenesis of ALS, and many clinical and animal studies have shown that complement factors, including C1q and C3, have a strong upregulation in the dead regions of motor neurons (90). HM has a complex mechanism of action and a wide range of effects. According to research, polysaccharides in natural HM are important components of HM to regulate complement activity (91). C1r, C1s, C3, C4 were the main targets of the crude polysaccharide extract of S. mellowsis, which inhibited the activity of the complement system (92). The polysaccharide component PsEULl3 of Eucommia ulmoides has very high anti-complement activity against the classical pathway (53). The APS-2 glucomglycan isolated and purified from the plant has good anti-complement activity, and its targets are Clq, C2, C3, C5 and C9 (54). Lentinan can decompose complement C3 into allergen C3a, the mechanism of which may be that the recognition spot on complement protein can recognize the structure of polysaccharide and activate the complement system. The main targets of quercetin 7,3’,4’ -trimethyl ether from patchouli were C1q, C2, C5 and C9 (55). We often know that Chinese herbs often act on the classical pathway of complement to inhibit the activity of the complement system, but in addition, the active ingredients of Chinese medicines can also act on the side pathway. Among them, C1q, C2, C4 and C9 are the main targets of the charming chun components extracted from Knotweed (56), The extract components can be adjusted by acting on different complement targets or multiple targets together (57). The (+) -catechin-3-0-b-D-1 (2-cinnamyl) -glucoside isolated and identified from the Chinese herb Anagardia showed different degrees of inhibition on the classical and bypass pathways of the complement system (58).

##### 3.2.4.4 Regulates T lymphocytes

In the central nervous system, CD4+T cells are believed to have neuroprotective effects, which can promote neuroprotection of glial cells and delay the progression of diseases by changing the morphology of glial cells (93). CD4+T cells can differentiate into regulatory and effector T cells, with the former regulating the proliferation of the latter. In the T-cell-mediated immune response, the imbalance of effector and regulatory T cells will trigger neuroinflammation and eventually lead to neuronal degeneration and necrosis (94). Regulatory T cells are responsible for regulating the immune response of the body, and usually play an important role in maintaining self-tolerance and avoiding excessive immune response damage to the body. In the early stage of ALS, the level of regulatory T cells increases and thus plays an anti-inflammatory role in the central nervous system, while in the late stage of rapid progression, the level of regulatory T cells decreases and shows the deterioration of neuroinflammation. Effector T cells are cells that proliferate and differentiate after T cells receive antigen stimulation, and have the function of releasing lymphocytes and can actively respond to stimulation. Observation of patients with ALS also showed that increased effector T cells in blood and cerebrospinal fluid were associated with decreased survival, while increased regulatory T cells in blood were associated with improved survival (59). Trichosanthin extracted from trichosanthes trichosanthes can directly increase the number of regulatory T cells, the level of immune index interleukin-10 and induce higher expression of forked head spiral transcription factor 3, thereby enhancing the immune regulatory ability of regulatory T cells (60). Dichloromethylamine, a soluble component of wheat zhai, can change AKT phosphorylation signaling, reduce the differentiation of helper T cells 17, and induce the proliferation of regulatory T cells to maintain balance (95). Zuogui pill can also up-regulate the immune index interleukin 10, so as to improve the immune response function of regulatory T cells (96).

##### 3.2.4.5 Regulates natural killer cells

Natural killer cells are key components of adaptive immunity and active cells with significant toxicity. One study showed an increase in NK cells in the blood of ALS patients compared to controls (61). At present, there are few studies on the relationship between NK cells and the occurrence and development of ALS, and the relationship between the two is unclear. However, infiltration of NK cells and increased expression of NKG2D ligands on MN were found in the motor cortex and spinal cord of deceased ALS patients, and NK cells were toxic to MN expressing NKG2D ligands. NK cells also secreted IFN-γ to activate microglia and damage regulatory T cells in the spinal cord of mSOD1 mice (62). These results suggest that NK cells may affect the occurrence and development of ALS through multiple immune mechanisms. Futenge mixture, which is composed of Futenge, Astragalus and red ginseng, can up-regulate CD2, CD95, PD-1 receptors and activated molecules on the surface of natural killer cells, and play an excellent regulatory role on natural killer cells (64).

All the above studies have shown that by influencing related proteins such as atretic zonule 1 and atretic protein 5, reducing the production of pro-inflammatory factors and nitric oxide, acting on complement targets, and upregating cell surface receptor molecules, HM can regulate the stability of the blood-brain barrier, inhibit the activation of microglia and complement system, weaken the toxicity of natural killer cells, and regulate the function of T cells. Thereby alleviating and treating ALS at the level of immune function (**Table 4**).

**Table 4:** The pathological mechanism of ALS.

HM	mechanism of action	immunologic mechanism
Borneol, milkvetch root	ZO-1, Claudin-5↑	Improve the tight connection structure and reshape the blood-brain barrier
Borneol with safflower	MMP-2, MMP-9 ↓; ZO-1, Claudin-5↑	
Sijunzi decoction	occlusal protein, ZO-1, closure protein 1 ↑	
Buyang huanwu decoction	vWF, VEGF, MMP-9, MMP-2↓	
Ginsenoside Rg1	ZO-1, occlusion-protein↑; MMP-2, MMP-9 ↓	
Breviscapine	CD63, claudin5, occludin, ZO-1↑	
Tripterine	The phosphorylation of ERK1/2 and NF-κB ↓; NO ↓	Inhibit microglia activation
Melittin	P38 mitogen-activated protein kinase↓	
Human placenta	NO synthase, cyclooxygenase 2 ↓	
Cape jasmine fruit, Polygala	TLR4↓	
KCHO—1	gp91phox subtype of NADPH oxidase, iNOS, P38MAPK, ERK1/2 phosphorylation↓	
Calycosin	TNF-α	
Barbated skullcup herb	C1r, C1s, C3, C4	Inhibit abnormal activation of complement system
Cablin patchouli herb	C1q, C2, C5, C9	
Tokyo violet herb	Different complement targets or multiple targets	
Dolomiaea costus	Clq, C2, C3, C5, C9	
Polygonum cuspidatum	C1q, C2, C4, C9	
Trichosanthes kirilowii	Tregs, Foxp3, IL- 10↑	Regulates T cell function
Fringed pink	Th17 cells ↓; Treg↑	
Zuogui pill	IL-10↑	
Futeng Mixture	CD2, CD95, PD1 receptor ↑	Regulates natural killer cells

ERK1/2, Extracellular signal-regulated kinase 1/2; NF-κB, Nuclear factor kB; P38MAPK, P38 mitogen Activated protein kinase; ZO-1, zonula occludens protein 1. Claudin - 5,closed for protein; MMP, Matrix metalloprotein Enzyme; JNK, C - Jun amino terminal kinase; COX-2, cyclooxygenase 2; INOS, Induction type Nitric oxide synthase; Foxp3, Fork head screw transcription factor 3; Tregs, Regulatory T cells; Occludin, Occludin protein.; NO, Nitric oxide.

## 4 Discussion

### 4.1 Summary of results

ALS poses a significant challenge to the medical community due to its nature as a neurodegenerative disease. Adjunct therapies, such as HM treatment, have garnered attention as potential avenues for novel therapeutic approaches, particularly in efforts to prolong the progression of ALS. This study aims to investigate the efficacy of HM therapy in an ALS mouse model, probing its potential mechanisms of action and its impact on immune system regulation.

In this meta-analysis, we synthesized data from 18 studies involving a total of 443 animals, aiming to evaluate the effects of herbal treatments on a mouse model of ALS. By amalgamating findings from these investigations, we gained a more comprehensive understanding of the potential of herbal therapy for ALS management. The research demonstrated significant positive effects of herbal treatments in ALS, highlighting the role of their active constituents in neurogenic regulatory pathways crucial for delaying neurodegeneration. Our meta-analysis results showed notable effects of herbal medicine in ALS mice. However, we also observed that the heterogeneity (I² value) in most analyses was quite high, suggesting significant variations in experimental design, animal models, treatment doses, and duration across the included studies. This heterogeneity may impact the stability and interpretability of the meta-analysis results. Therefore, caution is needed when interpreting these findings. To address the issue of heterogeneity, we employed a random-effects model to account for variability across studies, aiming to minimize the impact of these differences. Additionally, we conducted sensitivity analyses to assess the influence of individual studies on the overall results. Despite these efforts, it is important to acknowledge that high heterogeneity may reduce the generalizability of these findings. Despite these challenges, this study provides valuable insights into the potential of herbal medicine in ALS treatment. Herbal treatments may protect motor neurons and slow disease progression. However, future research should be conducted under more consistent study designs to reduce heterogeneity and further validate these findings.

The scoping review involved 35studies. Immune disorders play a crucial role in the pathological process of ALS. Studies have found that the activation of microglia and astrocytes in the central nervous system, as well as the increase in the number of pro-inflammatory peripheral lymphocytes and macrophages, directly affect the occurrence and progression of ALS. In particular, genetic mutations associated with ALS, especially SOD1, are thought to further increase neuroinflammation levels, further confirming the imbalance of the immune system in ALS pathophysiology. Clinical studies have not only revealed the influence of genetic variants on immune disorders, but have found that even in the absence of significant genetic changes, immune disorders lead to impaired function of regulatory T lymphocytes and increased proinflammatory macrophages. This further underscores the importance of the immune system in the onset and progression of ALS and underscores the need to consider immune regulation in treatment. In the future, developing effective methods to monitor the pathophysiology and progression of inflammation-mediated diseases will be important. Chemokines, especially CXC chemokines, may play an important role in the pathophysiology of ALS. Understanding their role in the onset and progression of ALS, as well as their modulated treatment of the disease, may provide new directions for the treatment of ALS.

HMs plays an important role in ALS treatment because of its immunomodulatory properties. Whether it is a single Chinese medicine or a compound Chinese medicine, ALS can be treated by protecting the blood-brain barrier, anti-neuroinflammation, inhibiting the activation of the complement system, regulating the toxicity of natural killer cells, and regulating the immune response mediated by T cells. Based on a bone marrow transplant experiment to evaluate the contribution of the immune system to movement disorders, specifically the role of CD8 T cells. One study concluded that pathological Senataxin expression in the hematopoietic system is necessary for the development of motor phenotypes in mice, supporting the idea that dysfunction of the nervous system and hematopoietic/immune system contributes to the onset or progression of ALS disease. While existing treatment options offer hope to ALS patients, there is a need to further delve into the mechanisms of immune disease and explore more personalized and integrated treatment strategies to address this challenging disease. By targeting treatments for diseases of the immune system, it is possible to slow disease progression, improve patients’ quality of life and extend their survival. These efforts will provide more hope and possibilities for future ALS treatments.

### 4.2 Study quality and risk of bias

Based on the evaluation results, the studies included in the review were assessed using CAMARADES and SYRCLE’s ROB tools to evaluate their methodological quality and risk of bias.

For CAMARADES, the quality scores of the studies ranged from 4 to 8, with a total score of 10. Specifically, one study received a score of 4; two studies received a score of 5; four studies received a score of 6; seven studies received a score of 7, and four studies received a score of 8. These scores indicate a moderate to high level of methodological quality in the included studies.

In contrast, for SYRCLE’s ROB, the quality scores of the studies ranged from 3 to 7, with a total score of 10. Two studies received a score of 3; five studies received a score of 4; seven studies received a score of 5; three studies received a score of 6, and one study received a score of 7. These scores suggest a moderate level of risk of bias in the included studies.

### 4.3 Analysis and discussion of therapeutic effect

Many studies on the efficacy of HM in treating ALS have highlighted affirmative results, with most studies yielding positive outcomes. This study conducted a meta-analysis of 18 animal studies investigating the efficacy of HM in treating ALS to provide a more objective and comprehensive evaluation. Among the included studies, a limited number were deemed high quality based on bias risk assessment. While two studies provided detailed descriptions of randomization using random number tables, the methods were unspecified in the remaining ten. Six studies did not specify the allocation concealment method, and the blinding implementation was only mentioned in three studies, with the remainder not addressing blinding. The primary outcome measures varied across the trials, with the majority utilizing onset time and survival time as endpoints; specifically, 11 out of 18 studies used onset time, while 16 used survival time. Some studies employed measures of motor function (e.g., stride length) and neuron-related indicators as efficacy measures, but the evaluation methods were inconsistent. Three studies reported disease duration in experimental animals as an outcome measure. A remarkable effects of HM in ALS mice, including onset time SMD=1.75, 95% CI (1.14 ~ 2.36), Z = 5.60, *P* < 0.01), survival time(SMD = 1.42, 95% CI (0.79 ~ 2.04), Z = 4.44, P < 0.01), stride length SMD=1.90, 95% CI (1.21 to 2.59), Z = 5.39, P < 0.01) and duration time MD=6.79, 95% CI [-0.28, 13.87], Z=1.88, P =0.06), showing HM’s certain efficiency in treating ALS mice.

### 4.5 Implication

This study employed a comprehensive multidimensional systematic evaluation approach to thoroughly investigate the efficacy of HM in treating ALS animal models. Through a comprehensive reanalysis of various outcome indicators, we aimed to provide evidence-based medical support for the clinical application of HM in ALS treatment. This study utilized a rigorous scientific statistical method—meta-analysis—which not only opens new avenues for clinical research on herbal medicine in our country but also promotes the integration of HM with modern evidence-based medicine.

Specifically, this study summarized recent research on HM treatment for ALS. Through systematic review, we analyzed the effects of HM on ALS onset time and survival time, identifying its potential efficacy in delaying disease progression and extending survival. However, while the systematic review provided overall efficacy data from preclinical studies, it could not fully elucidate the underlying biological mechanisms. Therefore, we conducted a scoping review and found that HM plays a significant role in immunotherapy for ALS. HM modulates innate and adaptive immune processes by reshaping the blood-brain barrier, inhibiting natural killer cell activity, suppressing complement system activation, regulating microglial cell activity, and restoring T cell function, thereby protecting motor neurons from toxic damage. These mechanisms not only provide a biological explanation for the efficacy observed in the systematic review but also deepen our understanding of the mechanisms through which HM acts in ALS treatment. These findings lay the foundation for further understanding the immunomodulatory mechanisms of HM in ALS treatment.

Combining the systematic review with the scoping review allowed us to delve deeper into the immune mechanisms of HM based on quantitative analysis of its efficacy. This dual-pronged research approach not only provides more comprehensive insights into current ALS research but also guides future clinical applications and drug development.

The significance of this study lies in its ability to deepen our understanding of HM in ALS treatment while also providing scientific evidence for the integration of modern evidence-based medicine with traditional herbal medicine. By reviewing the immunomodulatory mechanisms of HM in ALS treatment, we provide a theoretical basis and new therapeutic strategies for this refractory disease. Future research should continue to explore the potential role of HM in ALS treatment, aiming to bring more effective treatment options to ALS patients.

This comprehensive analysis not only enhances our understanding of ALS treatment methods but also advances the clinical application of HM in ALS, paving the way for research on refractory diseases. Through rigorous scientific research design and multidimensional evaluation methods, we provide a solid foundation for future ALS research and offer important references for the application of HM in modern medicine.

### 4.4 Limitations

Currently, Trials on the treatment of ALS with HMs are relatively scarce. However, our study faced some limitations, including the limitation of being limited to publicly published literature searches. This means that we may have missed some relevant grey literature that may contain important information on the effectiveness of HM in treating ALS. Second, our search covers only English and Chinese studies, which may introduce a degree of linguistic bias as studies in other languages may exist but are not taken into account. In addition, we need to recognize the existence of publication bias, that is, negative study results are relatively less likely to be published, which may cause our analysis to be influenced by positive studies and overestimate the therapeutic effect.

These limitations and biases may lead to varying degrees of bias in our findings. First, the incomplete coverage of the literature may mean that we miss some potentially important studies, resulting in an insufficiently comprehensive overall assessment of the efficacy of HM in the treatment of ALS. Secondly, due to the limited number and varying quality of studies, the results of our analysis may lack robustness and have certain uncertainties. In addition, due to the insufficient sample size, our analysis may lack the statistical power to draw accurate conclusions or generalize to the entire ALS patient population. Therefore, in order to more fully evaluate the efficacy of HM in the treatment of ALS, future studies need to overcome these limitations and biases. This could include expanding literature searches to include studies in grey literature and other languages, as well as strengthening assessments of research quality and controlling the effects of publication bias. At the same time, efforts should be made to increase the sample size and improve the quality of studies to ensure more reliable and robust analysis results and provide more convincing evidence support for the treatment of ALS with HM. Thirdly, A limitation of our study is the lack of detailed exploration of the side effects of HMs. Since our study focused on a preclinical systematic review, the issue of side effects of HM is rarely addressed in the existing literature. Detailed side-effect studies usually need to be performed in clinical studies. Considering the herbal medicine is widely used in the treatment of understanding the potential side effects is very important to ensure patient safety. We expect that future clinical studies will explore this issue in depth and provide more comprehensive and reliable data for systematic evaluation of the safety of Chinese herbal medicines.

## 5 Conclusion

The preclinical evidence supports the utilization of HM as a conventional treatment for ALS mice. Growing evidence indicates that HM may potentially delay neurological degeneration in ALS by activating diverse signaling pathways, especially immune pathways.

## Data Availability

The original contributions presented in the study are included in the article/**Supplementary Material**. Further inquiries can be directed to the corresponding author.

## Supplementary material

Supplementary Data Sheet 1 Specific search strategy.

Supplementary Data Sheet 2 Specific search strategy.
