# Supplementary material for: Herbal medicines for SOD1G93A mice of amyotrophic lateral sclerosis: preclinical evidence and possible immunologic mechanism
Source: Front Immunol. 2024 Sep 17;15:1433929. doi: 10.3389/fimmu.2024.1433929 (PMC11442286; doi:10.3389/fimmu.2024.1433929)
Supplement: Supplementary Data Sheet 1 — Specific search strategy. [file DataSheet1.docx]

Appendix 1

[Pubmed]

1. ("Amyotrophic Lateral Sclerosis"[MeSH Terms]) OR ("Motor Neuron Disease"[MeSH Terms]) 35205
2. (((((((((((("Amyotrophic Lateral Sclerosis"[Title/Abstract]) OR ("Lou Gehrig Disease"[Title/Abstract])) OR ("Lou Gehrig's Disease"[Title/Abstract])) OR ("Lou Gehrigs Disease"[Title/Abstract])) OR ("Gehrig's Disease"[Title/Abstract])) OR ("Gehrig Disease"[Title/Abstract])) OR ("Gehrigs Disease"[Title/Abstract])) OR ("Charcot Disease"[Title/Abstract])) OR ("Lou-Gehrigs"[Title/Abstract])) OR ("Motor Neuron Diseases"[Title/Abstract])) OR ("Motor Neuron Disease"[Title/Abstract])) OR ("Motor System Disease"[Title/Abstract])) OR ("Motor System Diseases"[Title/Abstract]) 35308
3. 1 OR 2 47620
4. ((((("Herbal Medicine"[MeSH Terms]) OR ("Drugs, Chinese Herbal"[MeSH Terms])) OR ("Medicine, Korean Traditional"[MeSH Terms])) OR ("Medicine, East Asian Traditional"[MeSH Terms])) OR ("Medicine, Chinese Traditional"[MeSH Terms])) OR ("Medicine, Kampo"[MeSH Terms]) 75459
5. ((((((((((((((((("Herb"[Title/Abstract]) OR ("Herbal"[Title/Abstract])) OR ("Herbal Medicine"[Title/Abstract])) OR ("Traditional Medicine"[Title/Abstract])) OR ("Oriental Medicine"[Title/Abstract])) OR ("East Medicine"[Title/Abstract])) OR ("East Medicines"[Title/Abstract])) OR ("East Asia Medicine"[Title/Abstract])) OR ("East Asia Medicines"[Title/Abstract])) OR ("Chinese Herbal"[Title/Abstract])) OR ("Chinese Plant Extracts"[Title/Abstract])) OR ("Chinese Plant Extract"[Title/Abstract])) OR ("Chinese Drugs"[Title/Abstract])) OR ("Chinese Drug"[Title/Abstract])) OR ("Korea Medicine"[Title/Abstract])) OR ("Korean Medicine"[Title/Abstract])) OR ("Kampo"[Title/Abstract])) OR ("Kanpo"[Title/Abstract]) 89060
6. 4 OR 5 145196
7. 3 AND 6 101
8. Up to April 10, 2024

[CNKI]

1. TI=(肌萎缩性脊髓侧索硬化症 + 肌萎缩侧索硬化症 + 肌萎缩性侧索硬化症 + 肌萎缩侧索硬化 + 乙酰乳酸合成酶 + Amyotrophic Lateral Sclerosis + Lou Gehrig Disease + Lou Gehrig's Disease + Lou Gehrigs Disease + Gehrig's Disease + Gehrig Disease + Gehrigs Disease + Charcot Disease + Lou-Gehrigs + Motor Neuron Diseases + Motor Neuron Disease + Motor System Disease + Motor System Diseases) OR AB=(肌萎缩性脊髓侧索硬化症 + 肌萎缩侧索硬化症 + 肌萎缩性侧索硬化症 + 肌萎缩侧索硬化 + 乙酰乳酸合成酶 + Amyotrophic Lateral Sclerosis + Lou Gehrig Disease + Lou Gehrig's Disease + Lou Gehrigs Disease + Gehrig's Disease + Gehrig Disease + Gehrigs Disease + Charcot Disease + Lou-Gehrigs + Motor Neuron Diseases + Motor Neuron Disease + Motor System Disease + Motor System Diseases) 50642
2. TI=(草药 + 中草药 + 中药材 + 中药 + 中医药 + 中药治疗 + 韩国草药 + 韩药 + 汉方 + 汉方医学 + 日本汉方医学 + Herb + Herbal + Herbal Medicine + Traditional Medicine + Oriental Medicine + East Medicine + East Medicines + East Asia Medicine + East Asia Medicines + Chinese Herbal + Chinese Plant Extracts + Chinese Plant Extract + Chinese Drugs + Chinese Drug + Korea Medicine + Korean Medicine + Kampo + Kanpo) OR AB=(草药 + 中草药 + 中药材 + 中药 + 中医药 + 中药治疗 + 韩国草药 + 韩药 + 汉方 + 汉方医学 + 日本汉方医学 + Herb + Herbal + Herbal Medicine + Traditional Medicine + Oriental Medicine + East Medicine + East Medicines + East Asia Medicine + East Asia Medicines + Chinese Herbal + Chinese Plant Extracts + Chinese Plant Extract + Chinese Drugs + Chinese Drug + Korea Medicine + Korean Medicine + Kampo + Kanpo) 1158094
3. 1 AND 2 595
4. Up to April 10, 2024
