# Supplementary material for: Herbal medicines for SOD1G93A mice of amyotrophic lateral sclerosis: preclinical evidence and possible immunologic mechanism
Source: Front Immunol. 2024 Sep 17;15:1433929. doi: 10.3389/fimmu.2024.1433929 (PMC11442286; doi:10.3389/fimmu.2024.1433929)
Supplement: Supplementary file 3 [file DataSheet3.docx]

Note. Green, Low risk. Yellow, Uncertain risk. Red, High risk
